# Intrinsic Nucleic Acid Dynamics Modulates HIV-1 Nucleocapsid Protein Binding to Its Targets

**DOI:** 10.1371/journal.pone.0038905

**Published:** 2012-06-20

**Authors:** Ali Bazzi, Loussiné Zargarian, Françoise Chaminade, Hugues De Rocquigny, Brigitte René, Yves Mély, Philippe Fossé, Olivier Mauffret

**Affiliations:** 1 Laboratoire de Biologie et Pharmacologie Appliquée, Ecole Normale Supérieure de Cachan, Centre National de la Recherche Scientifique, Cachan, France; 2 Laboratoire de Biophotonique et Pharmacologie, Centre National de la Recherche Scientifique Unité mixte de Recherche 7213, Faculté de Pharmacie, Université de Strasbourg, Illkirch, France; Institut Pasteur, France

## Abstract

HIV-1 nucleocapsid protein (NC) is involved in the rearrangement of nucleic acids occurring in key steps of reverse transcription. The protein, through its two zinc fingers, interacts preferentially with unpaired guanines in single-stranded sequences. In mini-cTAR stem-loop, which corresponds to the top half of the cDNA copy of the transactivation response element of the HIV-1 genome, NC was found to exhibit a clear preference for the TGG sequence at the bottom of mini-cTAR stem. To further understand how this site was selected among several potential binding sites containing unpaired guanines, we probed the intrinsic dynamics of mini-cTAR using ^13^C relaxation measurements. Results of spin relaxation time measurements have been analyzed using the model-free formalism and completed by dispersion relaxation measurements. Our data indicate that the preferentially recognized guanine in the lower part of the stem is exempt of conformational exchange and highly mobile. In contrast, the unrecognized unpaired guanines of mini-cTAR are involved in conformational exchange, probably related to transient base-pairs. These findings support the notion that NC preferentially recognizes unpaired guanines exhibiting a high degree of mobility. The ability of NC to discriminate between close sequences through their dynamic properties contributes to understanding how NC recognizes specific sites within the HIV genome.

## Introduction

The human immunodeficiency virus type 1 (HIV-1) nucleocapsid protein (NC) is a small (55 amino acids) basic protein characterized by two zinc fingers and a basic N-terminal domain [1], [2]. NC exhibits numerous functions all along the virus replication cycle [2]–[8], being notably involved in selective packaging of unspliced viral genomic RNA and chaperoning of nucleic acid strands during reverse transcription. The multiple roles of NC in virus replication are thought to result from its interplay with various target nucleic acid sequences [1]. At high concentrations, NC can bind non specifically to any DNA and RNA sequence of 5–7 nt length. In contrast, at low concentrations, the binding of NC strongly depends on the sequence and the structure of the DNA or RNA sequence [2], [9]–[11]. Numerous *in* *vitro* studies support the notion that NC zinc fingers are responsible for specific interactions, whereas the basic N-terminal domain is involved in non-specific binding [1], [2], [12]–[16]. A clear-cut feature of NC is its preference for single-stranded regions (bulges, loops, linear fragments,…) over double-stranded sequences and its ability to destabilize short double-strand regions. Interestingly, NC exhibits higher affinity for sequences containing unpaired guanines [2], [11]. More precisely, sequences containing TG, UG, and GNG (where N corresponds to either A, C, T or U) motifs are preferred [9]–[11], [17], [18]. High-resolution structures allow understanding the structural basis for this specificity, by showing that insertion of an unpaired guanine into the hydrophobic platform at the top of the folded zinc fingers is systematically present in all solved complexes. This insertion is thought to be critical for discriminating the guanine residue from the other bases [9], [11], [19]–[21].

In this context, one important question is to understand the molecular basis of the selective binding of NC to particular sequences, such as for instance the SL2 and SL3 stem-loops involved in the specific packaging of the unspliced viral RNA genome [11], [22]. The recent determination of the architecture and secondary structure of the entire HIV-1 RNA genome [23] indicates that large portions are double-stranded, suggesting that NC specific sites are limited. Furthermore, data with the Gag protein and the MuLV retroviral genome [24] indicate that the local context can considerably increase the NC affinity for particular sequences and show that a short motif (4 nt) with a low information content can be discriminated and identified in the entire viral genome. However, understanding of the molecular mechanisms involved in the selection process is limited and requires additional studies.

Using NMR methods, we recently investigated the binding of NC(11–55) to mini-cTAR, a model stem-loop DNA molecule of 26 nt [20], [25], that corresponds to the top half of cTAR, the complementary sequence of TAR (Trans activating response element) RNA [6], [14], [15], [26]–[28]. The annealing of cTAR with TAR is necessary for the first strand transfer of reverse transcription [6], [29]. The determination of the three-dimensional structure of mini-cTAR:NC(11–55) complex allowed by comparison with other reported NC:nucleic acid structures to identify the molecular determinants of the opposite binding polarity of NC on DNA molecules as compared to RNA molecules [20]. Interestingly, although five guanines of mini-cTAR are not involved in stable base pairing (defined on the basis of the presence or absence of a detectable imino proton signal at 10°C) in free cTAR and constitutes therefore potential binding sites, our NMR data indicate only one major binding site in mini-cTAR corresponding to the G26 residue of the ^24^TGG^26^ sequence at the 3′-end [20], [25]. Furthermore, all nucleic acid partners of NC used in the previous NMR studies had only one NC binding site [9], [11], [19], [21] and only in one case a minor binding site was identified besides the main site [19]. Therefore, the nearly exclusive binding of NC to the TGG sequence at the 3′-end of mini-cTAR is intriguing as is also the absence of significant binding to the apical and internal loops that contain unpaired guanines.

To further understand the origin of the preferential recognition of the TGG sequence by NC and the absence of significant binding in apical and internal loops, quantitative information on the motions experienced by DNA molecules in the presence and absence of NC are needed to complete the previous structural studies and to provide insights into the role of dynamics in the NC:DNA recognition. Although the top half of TAR RNA has been the subject of numerous NMR studies that describe the internal dynamics and relative motions of the stems of this hairpin [30]–[35], the cTAR element has been little studied with NMR methods. Here, using ^13^C spin relaxation, and relaxation dispersion measurements [35]–[39], we quantified the mini-cTAR DNA dynamics. Quantitative analysis of the relaxation data identified the main sites of the fast dynamical processes (in the ps-ns timescale) as well as the slow motions (in the µs-ms time scale). The relaxation rates and heteronuclear NOE have been measured for the C6, C8 and C1′ sites which allowed depicting the dynamics of residues at the level of both the bases and deoxyribose sugars. Large differences in the dynamics between the various parts of the hairpin were observed. Moreover, we identified several putative transient base pairs in the apical and internal loops and investigated their role in the stability of the different parts of the hairpin. Interestingly, due to these transient base pairs the unpaired guanines in the apical and internal loops and the lower stem are not fully accessible to interact optimally with NC. Therefore, only the guanines of the TGG sequence being not involved in transient base-pairs can constitute a strong binding site in this model sequence.

## Materials and Methods

### DNA Preparation

Uniformly ^15^N-^13^C enriched mini-cTAR DNA stem loop (26 nt) was obtained from SILANTES. For NMR studies, the labeled mini-cTAR (26) was dissolved in 300 µl (shigemi tube) of 13 mM Na/Na_2_(PO_4_) buffer (pH 6.5), 30 mM NaCl, and 0.2 mM MgCl_2_. After lyophilization, labeled mini-cTAR was dissolved in D_2_O. The final sample concentration of mini-cTAR was 0.75 mM. NC(11–55) was synthesized by the stepwise solid phase method with Fmoc amino acids as described [40] and its purity was greater than 98%. NC(11–55) was prepared with 3 equivalents of ZnCl_2_ to ensure saturation of the finger motifs.

### Gel Retardation Assays

Mini-cTAR DNA was 5′-end labeled using T4 polynucleotide kinase (New England Biolabs, Ipswich, MA) and [γ-^32^P] ATP (Perkin Elmer, Waltham, MA). The 5′-end labeled mini-cTAR was purified by electrophoresis on a 15% denaturing polyacrylamide gel and isolated by elution followed by ethanol precipitation. Assays were carried out in a final volume of 10 µl. Mini-cTAR ^32^P-DNA (10 pmol) at 2 × 10^3^ cpm/pmol was dissolved in 6 µl of water, heated at 90°C for 2 min and chilled for 2 min on ice. Then, 2 µl of renaturation buffer was added (final concentrations: 30 mM NaCl, 0.2 mM MgCl_2_ and 25 mM Tris-HCl pH 7.5) and the sample was incubated for 15 min at 20°C in the absence or presence of protein at various concentrations. Gel loading buffer (final concentrations: 10% w/v glycerol, 0.01% w/v bromophenol blue, 0.01% w/v xylene cyanol) was added and the samples were analyzed by electrophoresis on a 14% polyacrylamide gel (Acrylamide:Bis-acrylamide = 29:1) at 4°C in 0.5 × TBE buffer (45 mM Tris-borate pH 8.3, 1 mM EDTA). After electrophoresis, the gel was fixed, dried and autoradiographed. Free DNA and protein:DNA complexes were quantified using a Molecular Dynamics Typhoon imager and ImageQuant software (Molecular Dynamics, GE Healthcare Bio-Sciences Corp., Piscataway, NJ). The fraction of bound mini-cTAR DNA (FR) was determined using the formula FR = 1−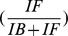, where IF and IB are the band intensities of free and bound mini-cTAR DNAs, respectively.

### NMR Relaxation Experiments

All NMR experiments were recorded at 298 K on Bruker Avance-500 spectrometer. Data were collected on BBI probes. Longitudinal relaxation rates (R1), transverse relaxation rates in the rotating frame 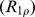 and heteronuclear NOE measurements were carried out for C8, C6 and C1′ carbons and were recorded as a series of 2D NMR spectra, in which the relaxation delay τ was parametrically increased. 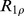 experiments were executed at a spin-lock field of 2 kHz with the carrier positioned at the center of the ^13^C resonance region. Since the different pairs of scalar coupled carbons resonate in distinct spectral regions, 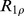 can be measured for C6 and C1′ without interference from unwanted magnetization transfers. Nonzero offsets of individual resonances cause measured 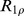 values to have contributions from R1 in addition to 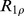. The true 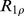 values were extracted according to: 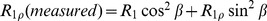 in which 
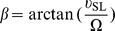
 is the effective tilt angle of the spin–lock field, with ν_SL_ and Ω being the spin-lock field strength and resonance offset in Hz respectively [38], [39]. The relaxation delay between scans was set to 2 s. The ^13^C carrier frequency was set to the center of the carbon resonance region of interest for the aromatic carbons and for the C1′. The ^13^C-^1^H correlation experiments were carried out with 86×2048 points with 48 scans for relaxation rate measurements on C6 and C8 carbons, whereas 120×2048 points were used for C1′ carbon with 48 scans.

Selective excitation was accomplished by application of ^13^C 180° IBURP1-shaped pulses during the first INEPT transfer to allow selective excitation of the described carbon resonances [41]. In addition, the ^13^C frequency labeling was implemented with a constant–time evolution period set to n/J_CC_ where J_CC_ is the scalar coupling between directly bonded carbons. The constant time delays were set to 7.10 and 12.5 ms when base and sugar relaxation properties were recorded, respectively, corresponding to ^13^C-^13^C coupling constants of approximately 70 Hz (bases) and 40 Hz (sugars). Delays of 5 ms, 20 ms, 60 ms, 100 ms, 200 ms, 400 ms, 700 ms, and 1000 ms; 4 ms, 8 ms, 12 ms, 24 ms, 32 ms, 48 ms, 64 ms, and 96 ms; 5 ms, 20 ms, 60 ms, 100 ms, 200 ms, 400 ms, 700 ms, 1000 ms respectively, were used for *T_1_* and *T_1_ρ* experiments of base ^13^C (C8 and C6) resonances. Several experimental points were repeated three times to evaluate the reproducibility of the measurements (60 ms, 200 ms and 700 ms for T_1_, 12 ms, 32 ms and 48 ms for T_1_ρ C8, and 60 ms, 200 ms, and 700 ms for *T_1_ρ* C6). Delays of 5 ms, 20 ms, 60 ms, 100 ms, 200 ms, 400 ms, 700 ms, 1000 ms, and 4 ms, 8 ms, 12 ms, 24 ms, 32 ms, 48 ms, 64 ms, 94 ms respectively, were used for *T_1_* and *T_1_ρ* of sugar (C1′) ^13^C resonances. Two times points in *T_1_ρ* were repeated (12 ms and 48 ms). Relaxation rates and errors due to uncertainty in experimental measurements were determined by fitting the intensity to a monoexponential decay using GraphPad Prism software. For heteronuclear NOE (hetNOE) measurements, a pair of spectra were recorded, one with initial proton saturation and one without. Spectra recorded with proton saturation utilized a relaxation delay of 2.5 s followed by a 2.5 s period of saturation. Spectra recorded in the absence of saturation employed a recycle delay of 2.5 s. Heteronuclear NOE spectra were collected at 500 MHz for the C6, C8 and C1′ resonances.

Data reporting on the power dependence of *T_1_ρ* were collected and analyzed for C8, C6 and C1′ resonances. A series of *T_1_ρ* experiments at various spin-lock field strengths was collected (0.9 kHz, 1.7 kHz, 2.6 kHz, 3.7 kHz, 5.2 kHz and 6.5 kHz). These values were set by modifying the power level of the spin-lock pulse. Data at each power level were collected and analyzed independently with the same settings and delays as those described above for *T_1_ρ* data collection. Delays of 4 ms, 8 ms, 12 ms, 16 ms, 24 ms, 32 ms, and 48 ms were used for *T_1_ρ* experiments for base ^13^C resonances. For sugars, delays of 12 ms, 20 ms, 28 ms, 40 ms, 60 ms and 80 ms were used. However, for the higher spin-lock fields (3.7 kHz, 5.2 kHz and 6.5 kHz), the longest relaxation delay was set to 28 ms to avoid sample heating, and delays were set to 4 ms, 8 ms, 12 ms, 16 ms, 24 ms, 28 ms for bases and 4 ms, 8 ms, 12 ms, 16 ms, 24 ms, 28 ms for sugars C1′.

### Data Analysis

Values of *T_1_* and *T_1_ρ* were determined by fitting the measured peak volumes for each assigned peak into an exponential decay. Briefly, T_1_ and T1ρ values were determined from the decay curves using the standard equation:
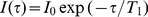
(1)where I_0_ is the initial peak intensity and τ is the delay time. The errors (S.D.) were estimated by the GraphPad Prism software. The hetNOE values were calculated from the ratio of the intensity of saturated/unsaturated spectra. Standard C-H bond lengths have been employed in all calculations. Commonly used C-H bond lengths are 1.08 Å for C6–H6/C8–H8 and 1.09 Å for C1′-H1′, respectively [42]. We used the values of chemical shift anisotropies (CSA) recently reported for C6/C8 (adenine 144 ppm, cytosine 186 ppm, guanine 133 ppm and thymine 168 ppm) [43], [44] while 30 ppm have been used for C1′ sugar [44], [45].

### Analysis of Relaxation Data with ModelFree

The model-free analysis of the relaxation parameters has been carried out using the Modelfree 4.0 program by Palmer and co-workers [46]. Modelfree parameters were fit to one of the five models, where the following parameters are varied: (1) S^2^, (2) S^2^ and an effective internal correlation time for fast motions τ_e_, (3) S^2^ and transverse relaxation exchange parameter R_ex_, (4) S^2^, τ_e_ and *R_ex_*, and (5) the order parameters for shorter and longer timescale motion (S^2^_f_, S^2^_s_,τ_f_,τ_s_).

For C6 and C8 carbon atoms, three residues (T10, C23 and T24) could be fit with model 2, two residues (G4, G8) were fit with model 3, five residues (C1, A5, C11, C12 and C22) with model 4 and the 11 remaining residues with model 1. For the anomeric resonances, four residues (G6, C9, T18 and C19) could be fit with model 1; three residues (A5, A7 and G20) could be fit with model 4. No residues were fit with model 3 and model 5. Residues C2, G4, T10, G14, A21, T24, G25 and G26 could not be fit with any model. C11 and C22 resonances overlapped. The nine remaining residues were fit with model 2.

## Results

### Analysis of the Sequence-specific Binding of NC to Mini-cTAR

**Figure 1:**
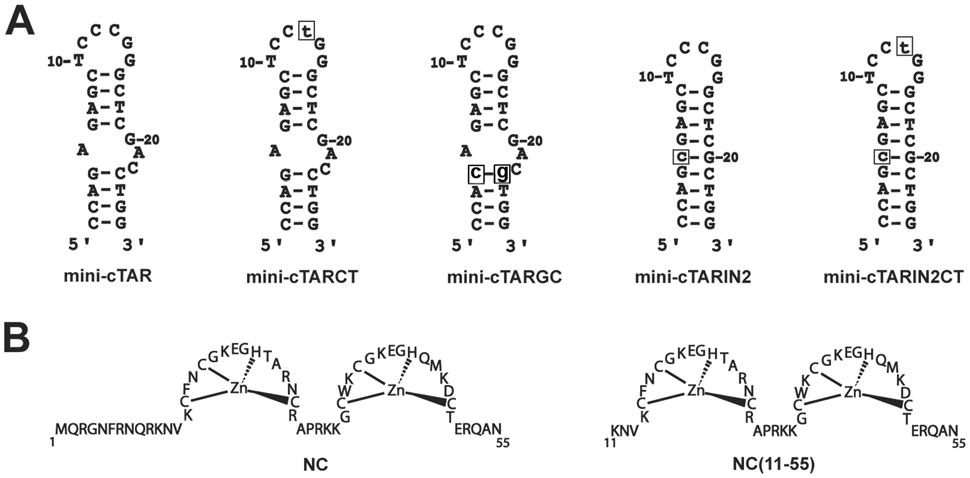
DNA oligonucleotides and peptides used in the study (A) Secondary structures for the mini-cTAR sequences. The single base mutations are boxed. (B) Sequences of NC and NC(11–55).

**Figure 2:**
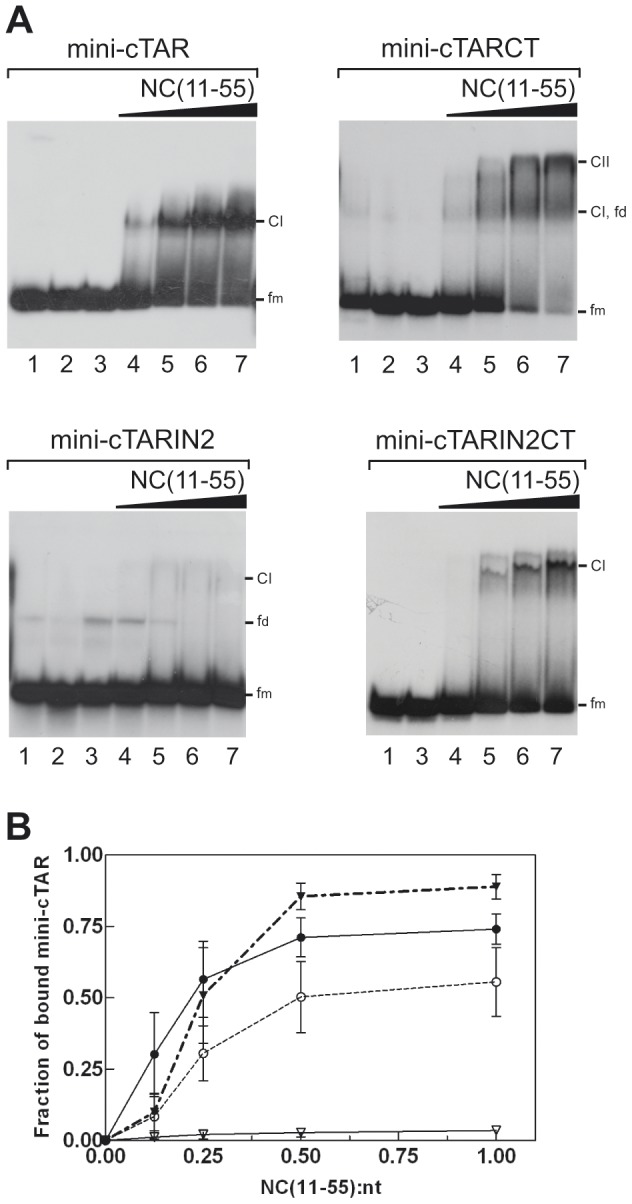
Gel retardation assays of NC(11–55):mini-cTAR DNA complexes formed *in vitro*. (A) Mini-cTAR ^32^P-DNAs were incubated in presence of NC(11–55) and analyzed by electrophoresis on a 14% polyacrylamide gel as described in Materials and Methods. Lanes 1, controls mini-cTAR dimerization induced by NC(11–55) at a protein to nucleotide molar ratio of 1∶1 (NC(11–55) was removed by phenol/chloroform before gel electrophoresis); lanes 2, heat-denatured mini-cTAR DNAs; lanes 3, controls without protein; lanes 4–7, protein to nucleotide molar ratios were 1∶8, 1∶4, 1∶2 and 1∶1. Monomeric and dimeric forms of free mini-cTAR DNAs are indicated by fm and fd, respectively. CI and CII indicate the NC(11–55):mini-cTAR complexes. (B) Fraction of bound mini-cTAR as a function of the protein:oligonucleotide (expressed in nt) ratio. Each data point represents the mean of three experiments. Symbols: filled circles, mini-cTAR; filled triangles, mini-cTARCT; open triangles, mini-cTARIN2; open circles, mini-cTARIN2CT.

The NC binding to mini-cTAR has been reported in our precedent NMR study [20]. We showed that the binding is almost restricted to a single site: the TGG sequence located at the 3′-end of the lower stem which alternates between single-stranded and double-stranded states [25], as a consequence of the destabilization induced by the internal loop. To gain further insight into the mini-cTAR DNA recognition by NC, we first carried out gel retardation assays with NC(11–55) (a truncated form of NC) and wild-type and mutant mini-cTAR DNAs (Figures 1 and 2). Interestingly, comparison with heat-denatured mini-cTAR DNAs (Figure 2A, lanes 2) revealed that the various mini-cTAR derivatives remained monomeric after renaturation and incubation with NC(11–55) at a protein to nucleotide molar ratio of 1∶1 and removal of the protein before gel electrophoresis (Figure 2A, lanes 1). Addition of increasing amounts of NC(11–55) to native mini-cTAR resulted in the appearance of band CI, consistent with the 1∶1 NC(11–55):mini-cTAR complex evidenced by NMR (Figure 2A). The IN2 mutant was designed so that the internal loop was deleted and the TGG sequence was predicted to be located in a stable double-stranded stem. Protein:DNA complexes were barely detectable with this mutant (Figure 2A and B), showing that NC(11–55) did not interact tightly with the apical loop of IN2, despite the presence of two unpaired guanines. The CT mutant was designed so that a single point mutation created a TGG sequence in the apical loop. Addition of increasing amounts of NC(11–55) resulted in the appearance of bands CI and CII, suggesting that the CT mutant contained two binding sites for the peptide: the TGG sequence at the 3′-end and the TGG sequence in the apical loop. The IN2CT mutant was designed so that the hairpin contained only one potential NC binding site corresponding to the unpaired TGG sequence in the apical loop. As expected, NC(11–55) bound mini-cTARIN2CT tightly with a 1∶1 stoichiometry, but the amount of protein:DNA complexes was less than with the wild-type (Figure 2B), indicating that the NC(11–55) binding strength depends on whether the TGG sequence is located at the 3′-end or in the apical loop. Therefore, the context of the unpaired TGG sequence appears important for NC(11–55) binding.

Taken together, one of the most intriguing features in our data was the poor binding ability of the CGG sequence in the apical loop of mini-cTAR. This may result from poor accessibility and availability of guanines, as a consequence of their dynamics in this context. To check this hypothesis, NMR ^13^C relaxation measurements were performed to probe the dynamic properties of the various residues of mini-cTAR with a particular focus on guanines.

### Data Collection and Qualitative Analysis

**Figure 3:**
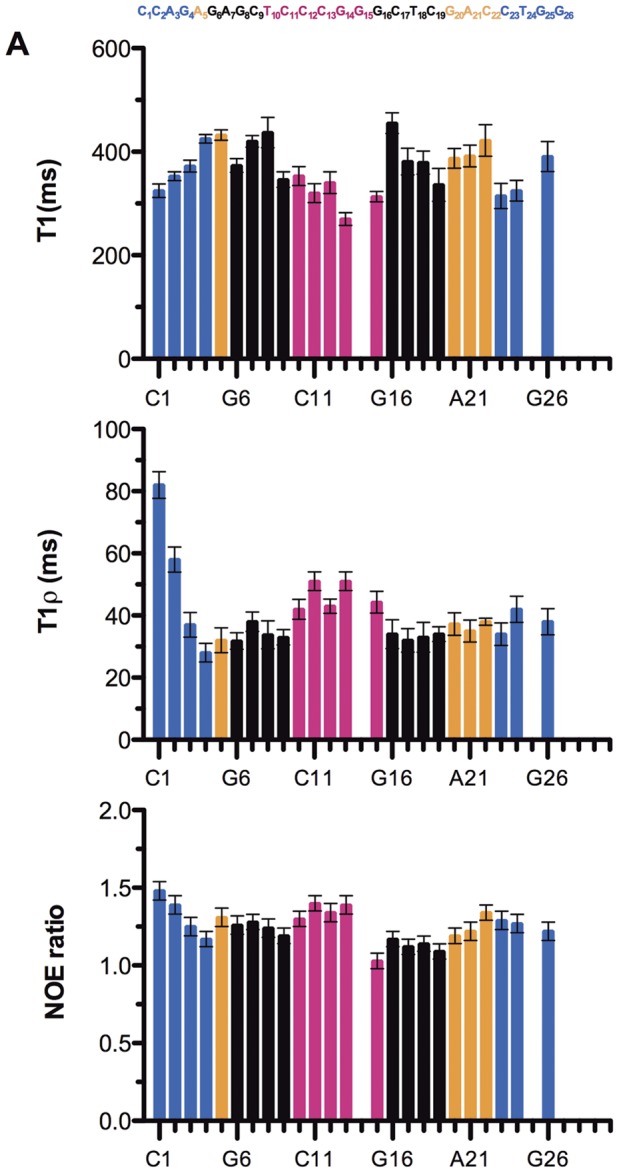
Relaxation times for aromatic C6/C8 spins of mini-cTAR DNA at 500 MHz. Top to bottom: ^13^C *T_1_*, ^13^C *T_1_ρ* and hetNOEs. Errors represent uncertainties in the fit of the primary relaxation data to mono-exponential decays. No data are associated to residues corresponding to a broad or overlapped cross peak. The color codes used for the residues are the following: blue (lower stem), orange (internal loop), black (upper stem) and magenta (apical loop).

To probe the dynamic properties of the mini-cTAR molecule, we recorded the ^13^C relaxation parameters of aromatic (C6, C8) and anomeric (C1′) carbons. Proton and carbon (C6, C8, C1′) resonance assignments for the mini-cTAR DNA residues were available from our previous study [25]. The ^13^C relaxation rates of C6, C8 and C1′ carbons were measured using a ^13^C, ^15^N-labeled sample of mini-cTAR. ^13^C, *T_1_*, *T_1_ρ* and hetNOEs were recorded for the aromatic and C1′ carbons using the constant time version of the pulse sequences modified for application to nucleic acids [41], [47], [48] as described in Materials and Methods. Typical *T_1_* and *T_1_ρ* relaxation decay curves are presented in Figure S1. Measured relaxation times *T_1_*, *T_1_ρ*, ^1^H-^13^C hetNOEs are shown in Figure 3 for C6 and C8 carbons, and in Figure S2 for C1′ carbons. To simplify the analysis, the mini-cTAR molecule was divided in four regions corresponding to the upper stem, lower stem, apical loop and internal loop (Figure 3). To compare the global properties of each part, we determined the average values for *T_1_*, *T_1_ρ* and hetNOE values for purine, pyrimidine bases and C1′ carbons for each part of the molecule (Table 1). Since only the upper stem is fully double-stranded [25], its values provided reference points to evaluate the relaxation properties of the other regions. For the upper stem, the relaxation parameters are ordered according the following hierarchy: *T_1_*__C1_′ (470 ms)>*T_1_*__purines_ (437 ms)>*T_1_*__pyrimidines_ (360 ms); *T_1_ρ*__C1′_ (44 ms)>*T_1_ρ*__purines_ (35 ms)>*T_1_ρ*_pyrimidines_ (33 ms); hetNOE__C1′_ (1.35)>hetNOE_purines (1.23)>hetNOE_pyrimidines (1.13). Interestingly, the hierarchy and the mean values are similar to those obtained for a DNA duplex [44] and for double-stranded regions in an RNA stem-loop [47], recorded at a similar frequency of 500 MHz. The relaxation differences between the three carbon classes (C8, C6 and C1′) are known to be related to large differences in chemical shift anisotropies between these three classes [47], [49]–[51].

**Table 1:** Average Relaxation times *T_1_*, *T_1_ρ* and NOE ratios for base and sugar resonances for each domain of mini-cTAR DNA.

	*T_1_* (ms)	*T_1_ρ* (ms)	NOE ratio
**Upper stem**			
C8–H8	437	35	1.23
C6–H6	360	33	1.13
C1′–H1′	470	44	1.35
**Lower stem**			
C8–H8	396	34	1.21
C6–H6	329	54	1.35
C1′–H1′	446	49	1.54
**Internal loop**			
C8–H8	403	34	1.24
C6–H6	421	38	1.34
C1′–H1′	471	37	1.36
**Apical loop**			
C8–H8	313	44	1.03
C6–H6	320	47	1.35
C1′–H1′	428	44	1.48

Relative to the upper stem, lower values for *T_1_* and higher values for *T_1_ρ* and hetNOE were observed for most residues of the apical loop and the lower stem (Table 1 and Comments on Figure 3 and Figure S2); suggesting the presence of fast motions on the picosecond to nanosecond time scale in these two parts of mini-cTAR [47], [52]. The average values for the internal loop do not show similar behavior, suggesting limited motions for this loop in the same time scale. Altogether, these data are consistent with our previous structural study indicating that, globally, residues of the apical loop and of the lower stem are poorly structured [25].

### Quantitative Analysis of the ^13^C Relaxation Data

Besides the qualitative features exposed above, the data were analyzed to get further information about the global motion, fast internal motions and slow conformational fluctuations of mini-cTAR. The procedure has been described extensively in previous works [41], [46], [53] and additional details are furnished as Materials S1 that were fitted with both axially symmetric and isotropic diffusion models and few differences were found between the residuals of the fits of the two models. The value for the global correlation time (5.1 ns) is close to that obtained for RNA molecules of similar size [47], [51]. The value of diffusion anisotropy 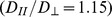 is slightly higher than 1 and indicates that the mini-cTAR structure did not present an elongated form [47]. This is in line with previous findings indicating that only the upper stem is stably formed, resulting probably in a non-elongated shape for mini-cTAR [25]. In contrast, the structure of the top half of the TAR RNA hairpin has been shown to be highly anisotropic with a 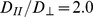 [54], consistent with the presence of two stems; each stem being composed of 4–6 base-pairs and separated by a three-base bulge. Another case that could be compared with the present data is the 23-nucleotide SRE, an RNA hairpin containing a long stem of 9 base-pairs, and for which a 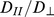 value of 1.49, indicating a significant degree of anisotropy, has been measured [51].

### Internal Motions

To extract information on the internal motions of individual residues, the relaxation data (*R1, R1ρ*, hetNOEs) were analyzed for the residues for which a complete set of measurements can be made. The relaxation data were analyzed with five models of different complexities: (1) S^2^; (2) S^2^, τ*_e_*; (3) S^2^, *R_ex_*; (4) S^2^, τ*_e_*, *R_ex_;* (5) S^2^f, S^2^, *τ_e_* using the model-free program [46], [55] to select the best model for each residue [42], [46], [47], [51], [54]. Several issues are known to complicate the analysis of ^13^C relaxation data for nucleic acids, such as the asymmetry of the chemical shift anisotropies, the non-colinearity of these latter with the C-H dipolar tensor, and the contribution to ^13^C relaxation from dipolar interaction with adjacent ^13^C and ^15^N spins [42], [47], [49]–[51], [54]. In the present case, these effects are expected to be weak considering the nearly isotropic behavior of cTAR, its small correlation time and the relatively average field (500 MHz) used for the study [47], [54].

**Figure 4:**
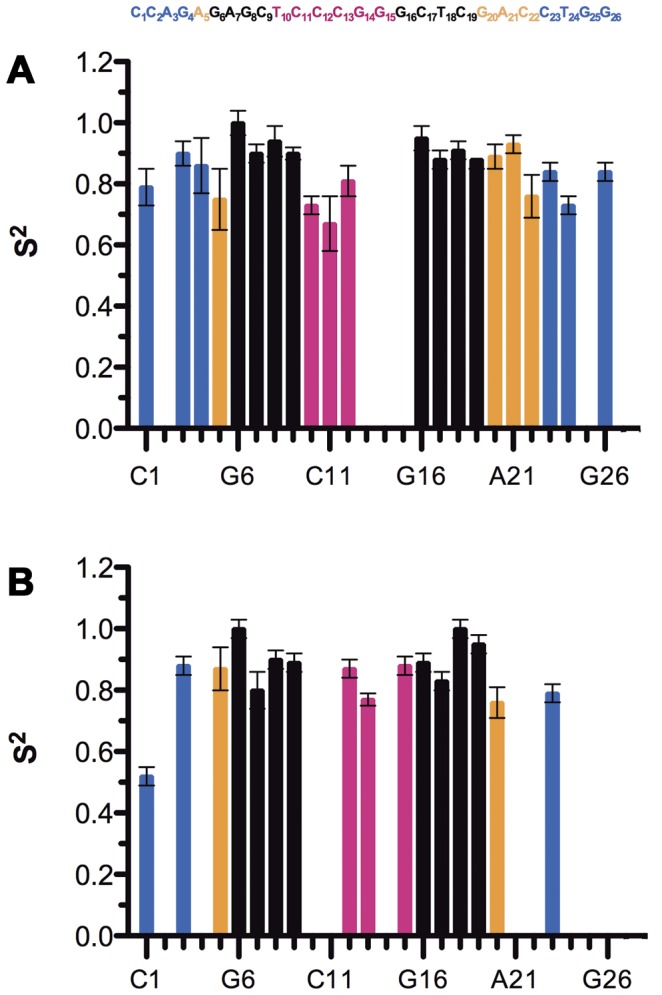
S^2^ as extracted from model-free analysis *versus* sequence at 500 MHz. (**A**) **C6/C8 spins;** (**B**) **C1′ spin.** Residues for which no results are shown correspond to overlapped cross-peaks or data that could not be fit well with any model-free model. The color codes used for the residues are the following: blue (lower stem), orange (internal loop), black (upper stem) and magenta (apical loop).

Results obtained for the different analyzed spins are reported in Table 2. For the aromatic carbons, most (7 out of 8) of the residues of the upper stem (G6–C9 and G16–C19) could be described by the most simple model (only the S^2^ parameter and internal fast time motions less than 20 ps) [46]. In contrast, the residues of the apical loop (T10–G15) are described by models 2 and 4 with correlation times for the internal motions in the range 15–100 ps. The internal motions of the internal loop residues G20 and A21 could be described by model 1 but more complex models were needed for A5 and C22 residues with correlation time in the range 40–65 ps and significant exchange contributions (*R_ex_*). Complex model are also necessary to describe the terminal C1, C23 and T24 residues of the lower stem. The S^2^ values are reported in Figure 4 (S^2^ ranges are between 0 (unrestricted motion) and 1 (highly restricted motion)). The upper stem residues (Figure 4A) show restricted motion (S^2^ in the range 0.88–1) consistent with the double-strand character of this part of mini-cTAR [25]. Lower S^2^ values (S^2^ in the range 0.67–0.81) indicative of less restricted motions are found for the pyrimidine residues of the apical loop (Figure 4A), consistent with the weak stacking interactions of these residues in the structure [25]. For the anomeric carbons, the differences in S^2^ are less significant between the residues of the different parts of the molecule, but the trends are similar (Table 2 and Figure 4B) since for instance the C12, C13 and G20 residues of the apical and internal loops exhibit lower S^2^ values than the residues of the upper stem (0.8–1.0). The residues of the lower stem were more difficult to fit since no model could describe the relaxation data of T24, G25, and G26. Finally, while ^1^H-^13^C aromatic cross-peaks of G25 and G26 overlap partially, ^1^H-^13^C1′ anomeric cross peaks for these two residues are clearly separated and intensities could be measured. Interestingly, while we could not fit the data of G25 and G26 to one of the models, the *T_1_ρ* and hetNOE values are higher for G26 than for G25 (Figure S2), suggesting higher mobility for G26. The large differences in the internal motions of residues from the different parts of the molecule are in full agreement with the data from our structural study [25].

**Table 2:** Internal motion parameters for mini-cTAR DNA. Due to overlapped cross-peaks and a broad peak, no models were fitted for C11, C22 sugars residues and for G14 base, respectively.

	C8/C6			C1′		
	S^2^	τ_e_(ps)	*R_ex_*(s^−1^)	S^2^	τ_e_(ps)	*R_ex_*(s^−1^)
C1	0.79±0.06	17.72±4.75	16.20±1.79	0.52±0.03	114.52±15.03	
C2						
A3	0.90±0.04			0.88±0.03	184.75±93.77	
G4	0.86±0.09		9.45±3.24			
A5	0.75±0.16	41.67±1.30	7.63±0.23	0.87±0.07	98.10±5.00	6.06±2.28
G6	1.00±0.04			1.00±0.03		
A7	0.86±0.03			0.80±0.06	41.22±18.29	4.61±2.21
G8	0.94±0.05		3.12±0.23	0.90±0.03	148.60±18.23	
C9	0.90±0.02			0.89±0.03		
T10	0.73±0.03	19.23±1.27				
C11	0.67±0.09	39.83±10.55	20.56±3.18	*Overlap*		
C12	0.81±0.05	97.23±39.86	2.23±0.12	0.87±0.03	154.30±68.57	
C13				0.77±0.02	252.22±43.34	
G14						
G15				0.88±0.03	221.76±176.86	
G16	0.95±0.04			0.89±0.03	81.65±43.45	
C17	0.88±0.03			0.83±0.03	94.32±13.23	
T18	0.91±0.03			1.00±0.03		
C19	0.88±0.03			0.95±0.03		
G20	0.89±0.04			0.76±0.05	29.40±12.31	8.43±1.74
A21	0.93±0.03					
C22	0.76±0.07	65.89±27.90	8.36±2.98	*Overlap*		
C23	0.84±0.03	74.84±37.68		0.79±0.03	254.73±69.54	
T24	0.73±0.03	41.43±15.99				
G25						
G26	0.84±0.03					

Other residues without values correspond to residues that do not fit any model well.

### Slow Conformational Exchange Fluctuations

**Figure 5:**
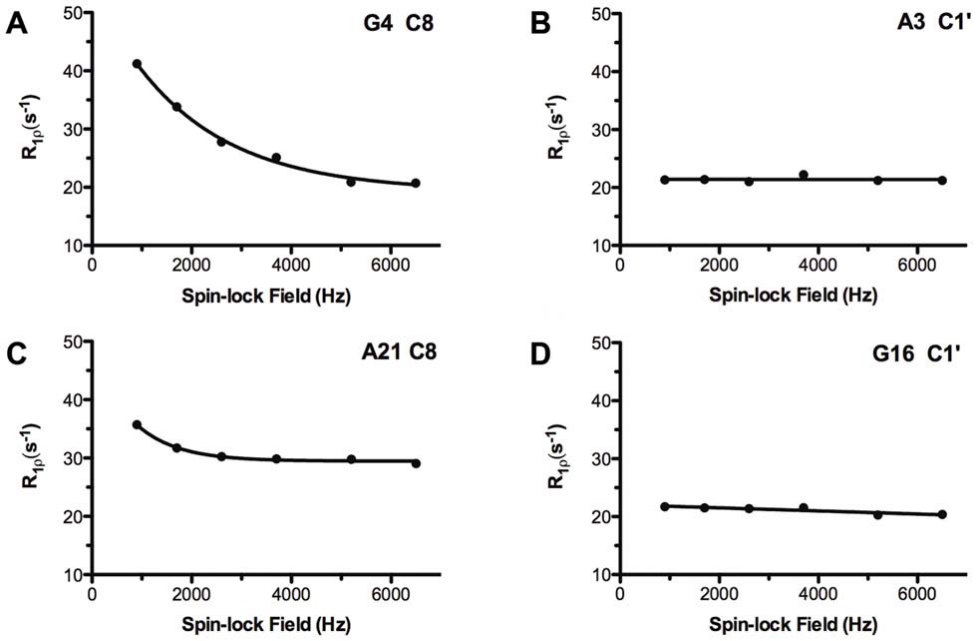
^13^C transverse relaxation rates (*R_1_ρ*) versus applied spin lock field strength (in Hertz) at 500 MHz for aromatic and anomeric carbons of mini-cTAR DNA. (A) G4 C8; (B) A3 C1′; (C) A21 C8; (D) G16 C1′.

**Figure 6:**
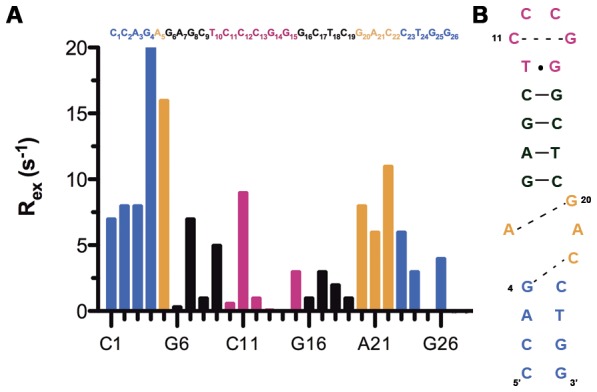
Exchange contribution to transverse relaxation versus sequence for aromatic C8 and C6 carbons (A) of mini-cTAR DNA determined from *T_1_ρ* power dependence experiments. (B) Secondary structure for the mini-cTAR sequence showing the possible transient base-pairs for G4–C22, A5–G20 and C11–G14. The color codes used for the residues are the following: blue (lower stem), orange (internal loop), black (upper stem) and magenta (apical loop).

The presence of slow motions in the µs-ms range led to an increase in the transverse relaxation rate by a factor *R_ex_* [39], [46], [56]. As a consequence, resonances exhibiting slow motions show a decrease in *R_1_ρ* as the power of the spin-lock field is increased. This power dependence of *R_1_ρ* for the aromatic and anomeric carbons of several residues is shown in Figure 5 and the *R_ex_* values that could be deduced from these curves are shown in Figure 6 for the aromatic carbons. The residues A5, G20 and C22 of the internal loop and the adjacent G4 residue present large slow exchange contributions. In contrast, in the upper stem and the apical loop, small exchange contributions are observed, with the exception of residue C11. Residues C1–A3 and T24–G26 of the lower stem present medium *R_ex_* values. The model-free analysis described in the preceding section can also be used to extract *R_ex_* values independently from the dispersion relaxation experiments. Comparison of the data with the two methods establishes that G4, A5 and C22 exhibit the largest *Rex* values (Table 2).

The data from anomeric carbons confirm the large exchange contributions for residues G4, A5, G20 and C22. Using both dispersion relaxation experiments and model-free analysis, we observe that the residues in the double-stranded part of mini-cTAR are devoid of significant exchange contributions and constitute therefore excellent controls for the reliability of the experiments. In the case of the C8 carbon of G14 residue, the corresponding cross-peaks are so broadened that it is not possible to reliably measure its intensity (it is the lowest intensity cross-peak in Figure S3). Such behavior is typical of large *Rex* contribution and slow exchange conformational fluctuations that are well known to impact directly the line width. Although the relaxation parameters could not be measured for the aromatic carbon of residue G14, it was possible to deduce from the recorded spectra that this residue possesses a large *Rex* value.

Taken together, the dispersion relaxation experiments, model-free analysis and qualitative observation of line broadenings demonstrate that four residues of the internal loop (A5, G20, A21 and C22), two residues of apical loop (C11 and G14) and the adjacent residues of the lower stem (G4, C23) exhibit slow conformational exchange fluctuations. In contrast, no residue of the upper stem is affected by these exchange contributions, demonstrating that the internal loop destabilizes only the lower stem but not the upper stem.

### Impact of Mutations in the Lower Stem

**Figure 7:**
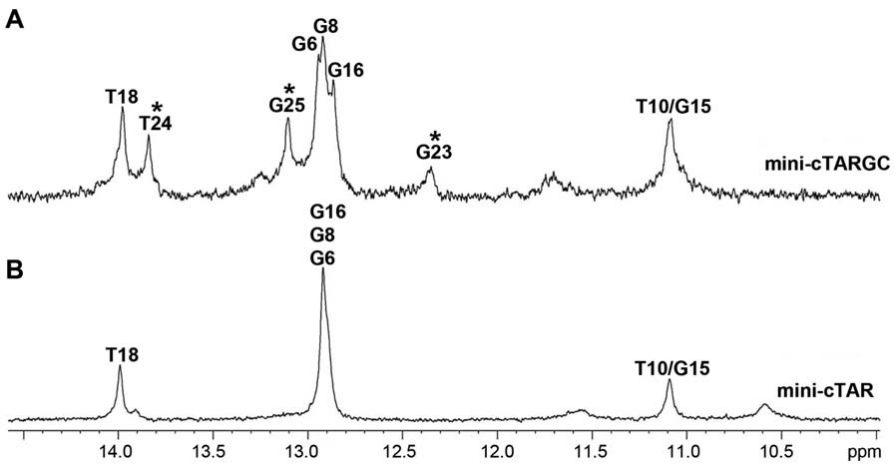
Imino region of 1D spectra (pH 6.5, in H_2_O, 10°C, 60 ms) showing the difference between mini-cTARGC (A) and mini-cTAR (B). The new resonances observable in the mutant and corresponding to the imino protons of the lower stem are indicated by asteriks.

To investigate the role of the junction between the internal loop and the lower stem on the stability of mini-cTAR, residues G4 and C23 were permuted in the mini-cTARGC mutant (Figure 1A). Interestingly, additional resonances, relative to mini-cTAR, were observed in the imino proton region (Figure 7). The procedure assignment based on the observation of NOE connectivities designate the new imino protons as those of residues G23 (broadened but clearly observable at 12.4 ppm), T24 and G25. The signal at 11.1 ppm in the two molecules has been assigned to the T10/G15 imino proton (Figure 7A and B). This latter assignment derived from the comparative analysis of a mutant of mini-cTAR in which residue G15 has been replaced by residue A15. The spectra of this mutant showed an additional T imino proton in the Watson-Crick region, as expected from the formation of the new T10–A15 base-pair and concomitantly the disappearance of the imino proton at 11.1 ppm (data not shown). In summary, the observation of three new imino protons in mini-cTARGC demonstrates that the permutation of the G4 and C23 bases strongly stabilizes the lower stem.

## Discussion

In this study, we investigated the motional properties of the residues of the top half of the cTAR DNA (mini-cTAR) by measuring the ^13^C relaxation properties of both aromatic and anomeric carbons. It is important to keep in mind that mini-cTAR is a model sequence that will not allow determining exactly how NC interacts with the full-length cTAR (55 nt). Indeed, the TGG motif identified as the main NC binding site in mini-cTAR is partly paired in the full-length cTAR [15], [25], and thus does likely not bind NC in this sequence. Nevertheless, due to its limited size, mini-cTAR constitutes a very good model system to investigate in depth by NMR the relationships between the dynamics of DNA residues and their recognition by NC. Knowledge of the dynamics of mini-cTAR residues is important to further understand the role of the dynamics of each nucleotide in the recognition mechanism of nucleic acids by NC and the role of the internal and apical loops on the stability of their adjacent stems. Mismatches, bulges and internal loops are regularly distributed along the TAR and cTAR hairpins delineating short double-stranded segments that can be easily melted by the weak duplex destabilizing properties of NC [2], [5], [6], [57], [58]. Moreover, the internal loop of mini-cTAR destabilizes the lower double-stranded segment [25] and favors NC binding [20] to the TGG sequence that constitutes the preferential binding site for NC(11–55) in this model sequence. In the mini-cTAR:NC(11–55), the T24 residue was found to interact with residues of the N-terminal zinc finger, while the G26 residue is inserted in the hydrophobic plateau of the C-terminal zinc finger, as in all published NC:nucleic acids complexes [11], [19], [21], [59], [60]. Using qualitative analyses of spectra and quantitative model-free analyses, we identified residues that are involved in: (i) slow motions (micro-to millisecond); (ii) fast motions (pico-to nanosecond); and (iii) the two types of motions, simultaneously. Our data are discussed in connection with our recent NMR study [20] and the gel retardation assays (Figure 2).

To gain insight into the recognition mechanism of DNA sequences by NC, it is important to understand why the G26 residue is selected in the context of mini-cTAR. Indeed, this hairpin contains five unpaired guanines: one in the apical loop (G14 since G15 is involved in a mismatch with T10), one in the internal loop (G20) and three in the destabilized lower stem (G4, G25 and G26). Among these potential binding sites, the strong preference exhibited for a single one is puzzling. Gel retardation data (Figure 2) showed that the apical loop does not significantly bind NC(11–55). Moreover, inclusion of the 3′-end TGG sequence within a stable stem (Figures 1 and 2, mutant mini-cTARIN2) precludes strong binding of NC(11–55), showing that the 3′ terminal position of G26 in mini-cTAR is not sufficient for NC(11–55) binding. This finding is consistent with data obtained with other oligonucleotides containing a guanine residue at the terminal position and that do not exhibit significant NC binding at this position [9], [11], [19]. For instance, the major binding site in the (-)PBS oligonucleotide is centered on a guanine located in the apical loop, whereas the guanine at the 5′ terminal position is only a minor binding site [19].

Several works clearly indicated that unpaired guanines are necessary for binding NC with high affinity, and the nature of the adjacent nucleotides is important too [2], [10], [18], [24]. Especially, the presence of a thymine at the 5′ side is a favorable factor, due to direct contacts between the methyl of the thymine residue and the hydrophobic side chains of the protein [19], [20]. To investigate the importance of this factor in the present case, we introduce the TGG sequence in the apical loop (Figure 1, mutants mini-cTARCT and mini-cTARIN2CT). The gel retardation experiments show a stronger binding of NC(11–55) to the mini-cTARIN2CT mutant exhibiting a TGG sequence in the apical loop, than to the mini-cTARIN2 mutant exhibiting a CGG sequence in the apical loop (Figure 2). Note that in these two mutants, the internal loop was deleted to prevent the NC binding to the TGG motif of the lower stem. Interestingly, the level of NC binding to the TGG motif is lower in the apical loop than in the lower stem (Figure 2B, compare mini-cTAR vs mini-cTARIN2CT). This is further supported by the data obtained with the mini-cTARCT mutant showing a moderate increase of NC binding relative to mini-cTAR, although this oligonucleotide contains two TGG sites: one in the lower stem and the other in the apical loop.

Thus, our data indicate that other factors than location of G26 at the 3′-end and the presence of a thymine at its 5′ side are necessary to fully explain the selective recognition of G26 in mini-cTAR. Our previous investigations on mini-cTAR alone identified slow dynamic processes in the apical and internal loops but not in the lower stem [20]. These data prompted us to hypothesize an important role of DNA dynamics in the mechanisms of NC:DNA recognition. To test this hypothesis, we investigated the DNA dynamics using ^13^C NMR relaxation.

### Selective Binding to NC Requires a Highly Mobile and Accessible Residue

Residues experiencing fast internal motions (low S^2^) were found to be located in the apical loop (T10, C11 and C12) and in the lower stem (C1, T24 and G26). Among the G residues, the exchange contributions found for G4, G14 and G20 indicate that these residues undergo stacking or hydrogen bonding interactions with neighbor residues. In contrast, residues G6, G8 and G16 in the upper stem did not present any exchange contribution supporting their inclusion in a stable double-strand structure. Similarly, residue G15 in the apical loop did not exhibit any exchange contribution indicating that it is likely paired with the T10 residue as confirmed by the disappearance of the T10/G15 imino proton (Figure 7) when residue G15 was replaced by A15 (data not shown).

For each residue exhibiting a significant exchange contribution, we search in its neighboring a possible partner residue experiencing chemical exchange contribution in the same range of time. For the G14 residue, no conformational exchange is found for its adjacent residues C13 and G15, suggesting that these residues do not interact with G14. However, significant exchange contributions are found for residue C11 both in dispersion experiments and in spin relaxation measurements (large *R_ex_* found for model 3), suggesting the existence of a transient C11–G14 base-pair (Figure 6). Interestingly, a similar cross-loop base-pair has been identified in the apical loop of TAR [52]. Noticeably, two residues separate the two partners of the C11–G14 base-pair in mini-cTAR DNA, instead of three for the apical loop of TAR RNA, suggesting that the C-G base-pair is less relaxed and stable in mini-cTAR than in TAR. Exchange contributions from dispersion relaxation measurements showed that A5 and G20 residues (Figure 6B) were involved in conformational exchange occurring in the same timescale. Therefore, a transient G.A base pair (of the shear type) likely occurs within the internal loop. This pairing, which frequently occurs in internal bulges [61], was already suggested by our structural studies [25]. Similar R_ex_ values were found for residue G4 in the lower stem and residue C22 in the internal loop indicating a possible transient pairing between these residues (Figure 6B), and thus, an alternate pairing of residue G4 with residues C22 and C23 that could explain the destabilization effect of the internal loop on the lower stem. This hypothesis is strongly supported by the analysis of the mini-cTARGC mutant (Figure 1A) that is unable to form the G4–C22 base-pair but exhibits three additional stable base-pairs in the lower stem (Figure 7). Similar base-pairs between residues of stems and internal loops have been described in the HIV-1 SL1 stem-loop [62]. Finally, residues G25 and G26 were the only G residues not involved in base-pairs (as assessed by the observation of imino protons) or in conformational exchange. Comparison of the respective dynamics of these two latter residues indicates a higher mobility of G26 relative to G25.

Taken together, our results strongly suggest that conformational exchange processes, probably associated to transient base pairing, are deleterious for NC recognition, explaining the low binding of the protein to the apical and internal loops and in contrast, its tight binding to G26 that is mobile and exempt of any pairing. In addition, we suggest that the lower binding of NC to the TGG motif in the apical loop (in mini-cTARCT and mini-cTARIN2CT) as compared to the TGG motif in the lower stem (mini-cTAR) is related to different dynamic processes. Of course, a full account of these effects would need a study of the dynamics of TGG inserted in the apical loop. Furthermore, it is important to note that the present study probes motions in two particular time-scales (the ps-ns scale from spin relaxation experiments and the µs-ms scale from the relaxation dispersion experiments). Therefore, we cannot rule out the possibility that intermediate ns-µs range motions may be also important for NC binding. Exploring motions in this time range would need site-specific deuterium labeling and solid-state NMR [63], [64], which are beyond the scope of the present study.

### Comparisons with Data from the Literature

Taken together, our NMR and binding data support the notion that residue G26 is not involved in a stable or transient base-pair and exhibits the highest mobility and accessibility from all the guanines present in mini-cTAR. The propensity of NC to bind preferentially a highly mobile G residue is further supported by several structural studies on oligonucleotides exhibiting a strong binding site for NC. Examination of the structures of free SL3 and SL2 [9], [65] that bind NC through the GNG motif in their apical loops [11], [60] shows that these guanines are totally looped out in solution in contrast to the other residues. Similarly, the structure of free PBS [19] that binds NC through the 5′-CTG-3′ motif of its loop shows also that the G7 residue is partially looped out with its Watson-Crick side directed towards the solvent. Moreover, the two guanines recognized by NC in the internal loop of SL1 are also highly mobile [62]. Furthermore, a high mobility of residue G32 in the apical loop of TAR RNA was recently reported [52] and is consistent with the specific interaction of NC with the apical loop of wild-type TAR RNA [14]. In contrast, the absence of binding of NC(11–55) to the apical loop of mini-cTAR DNA could be readily explained by the low mobility and accessibility of the G residues in this loop (this work).

### Conclusions

The present study investigates the molecular basis of the specific binding of NC to a particular guanine in a molecule possessing five unpaired guanines [20]. The local selection appears to depend on the relative mobility and accessibility of the residues and on the presence of a thymine at the 5′ side of the guanine residue. The high mobility of the G residue in the NC binding site is a direct consequence of the absence of a stable or transient base-pairing involving this residue. This is critical since the Watson-Crick side of the guanine was shown to interact with NC amino side chains and backbone atoms [11], [20]. In addition, it is likely that through its high mobility, the G residue can optimally adjust to the NC hydrophobic platform at the top of the folded fingers, and notably to the Trp37 residue, whose stacking with the G residue plays a key role in the binding energy [13], [66]. The key role of the oligonucleotide dynamic is also fully consistent with its role in the NC chaperone activity [67], [68]. The ability of NC to discriminate between close sequences through their dynamic properties contributes to understand how the NC domain of Gag recognizes the genomic RNA through specific interactions with the apical loops of SL2 and SL3.

## Supporting Information

Figure S1 **Representative decay curves for ^13^C relaxation experiments recorded at 500 MHz.** (A) ^13^C *T_1_* and (B) ^13^C *T_1_ρ* experiments for: A7 C8 (black circles), G16 C8 (red triangles) and T24 C6 (blue square) resonances. The results of duplicated experiments are included to confirm the reproducibility of the data. Most decay curves could be fitted with single exponentials.
(TIF)

Figure S2 **Relaxation times for anomeric C1′ carbons of mini-cTAR DNA at 500 MHz.** spins. Top to bottom: ^13^C *T_1_*, ^13^C *T_1_ρ* and hetNOEs. Errors represent uncertainties in the fit of the primary relaxation data to mono-exponential decays. No data are associated to residues corresponding to a broad or overlapped cross peak. The color codes used for the residues are the following: blue (lower stem), orange (internal loop), black (upper stem) and magenta (apical loop). ***Comments of*** ***Figure 3*** ***and Supplementary Figure S2***: The profiles of *T_1_*, *T_1_ρ* and hetNOE values for both C1′ and C6/C8 carbons were found to be correlated with the sequence. The *T_1_* profile for the successive parts of the molecule could be roughly described as: low (lower stem), high (upper stem), low (apical loop), high (upper stem), high (internal loop), low (lower stem). Note that for the *T_1_ρ* and hetNOE values, the profile is reversed. This profile is more apparent when only the central part (upper stem and apical loop) is considered. The data are therefore compatible with fast motions in the picosecond to nanosecond timescale for several residues of the apical loop. This kind of motion affects probably in an opposite way the *T_1_* (decrease) and *T_1_ρ*/hetNOE (increase) values [44], [47]. Similar profiles for the various parameters of aromatic and C1′ carbons are indicative of coupled motions for base and sugar of the various residues of mini-cTAR [47].
(TIF)

Figure S3 **Region C8/C6–H8 of constant–time HSQC of ^15^N/^13^C labeled mini-cTAR DNA at 30°C.** The cross peaks are indicated with the name of the corresponding residue. In this spectrum, G14 residue shows the broadest cross-peak among all residues of mini-cTAR (see text).
(TIF)

Materials S1 **Description of the procedure used in Quantitative Analysis of the 13C relaxation data.**
(DOC)
