# Supplementary figures and images for: Intrinsic Nucleic Acid Dynamics Modulates HIV-1 Nucleocapsid Protein Binding to Its Targets
Source: PLoS One. 2012 Jun 20;7(6):e38905. doi: 10.1371/journal.pone.0038905 (PMC3380039; doi:10.1371/journal.pone.0038905)

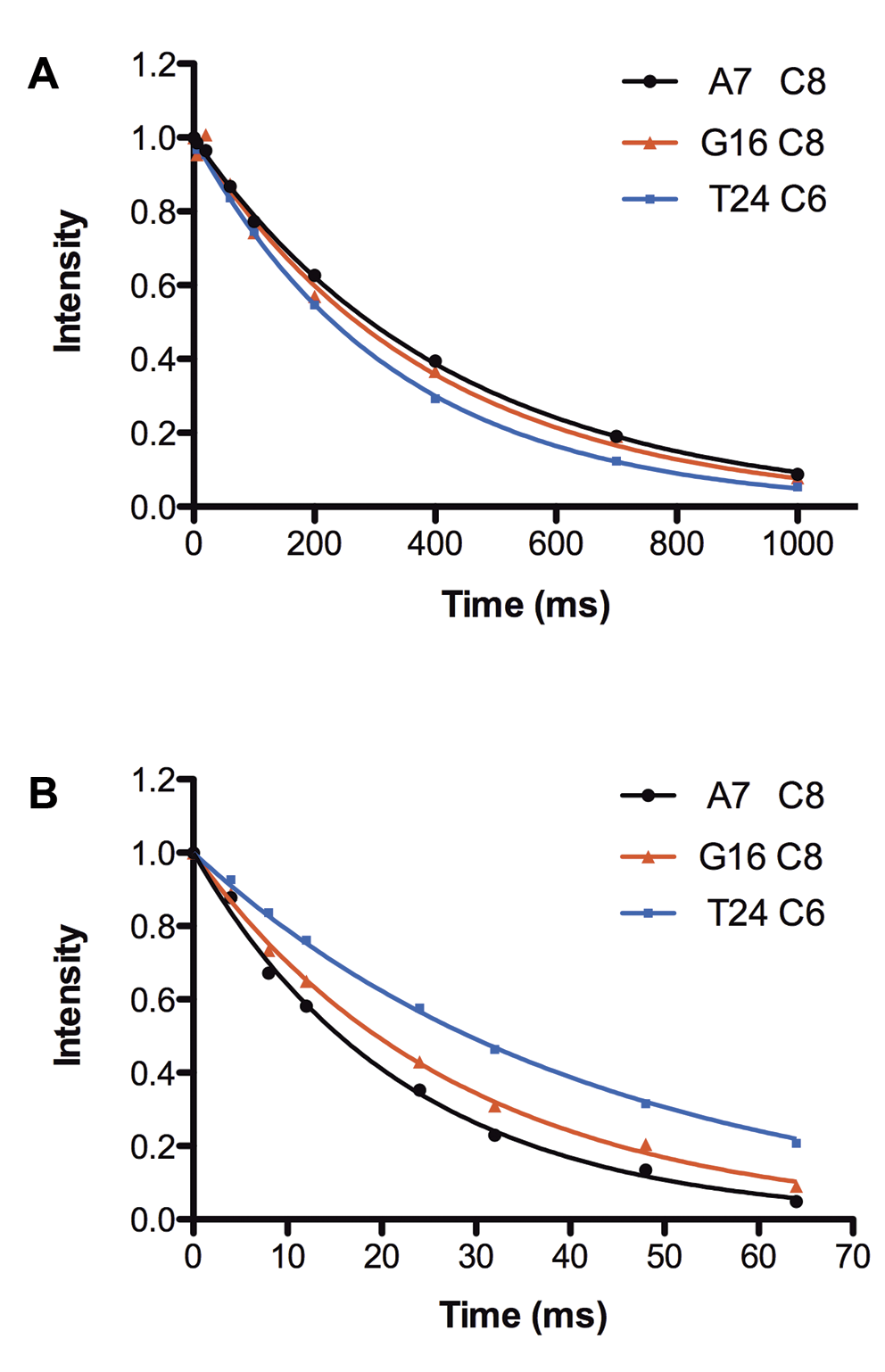

Supplement: Figure S1 — Representative decay curves for 13C relaxation experiments recorded at 500 MHz. (A) 13C T1 and (B) 13C T1ρ experiments for: A7 C8 (black circles), G16 C8 (red triangles) and T24 C6 (blue square) resonances. The results of duplicated experiments are included to confirm the reproducibility of the data. Most decay curves could be fitted with single exponentials. (TIF) [file pone.0038905.s001.tif]

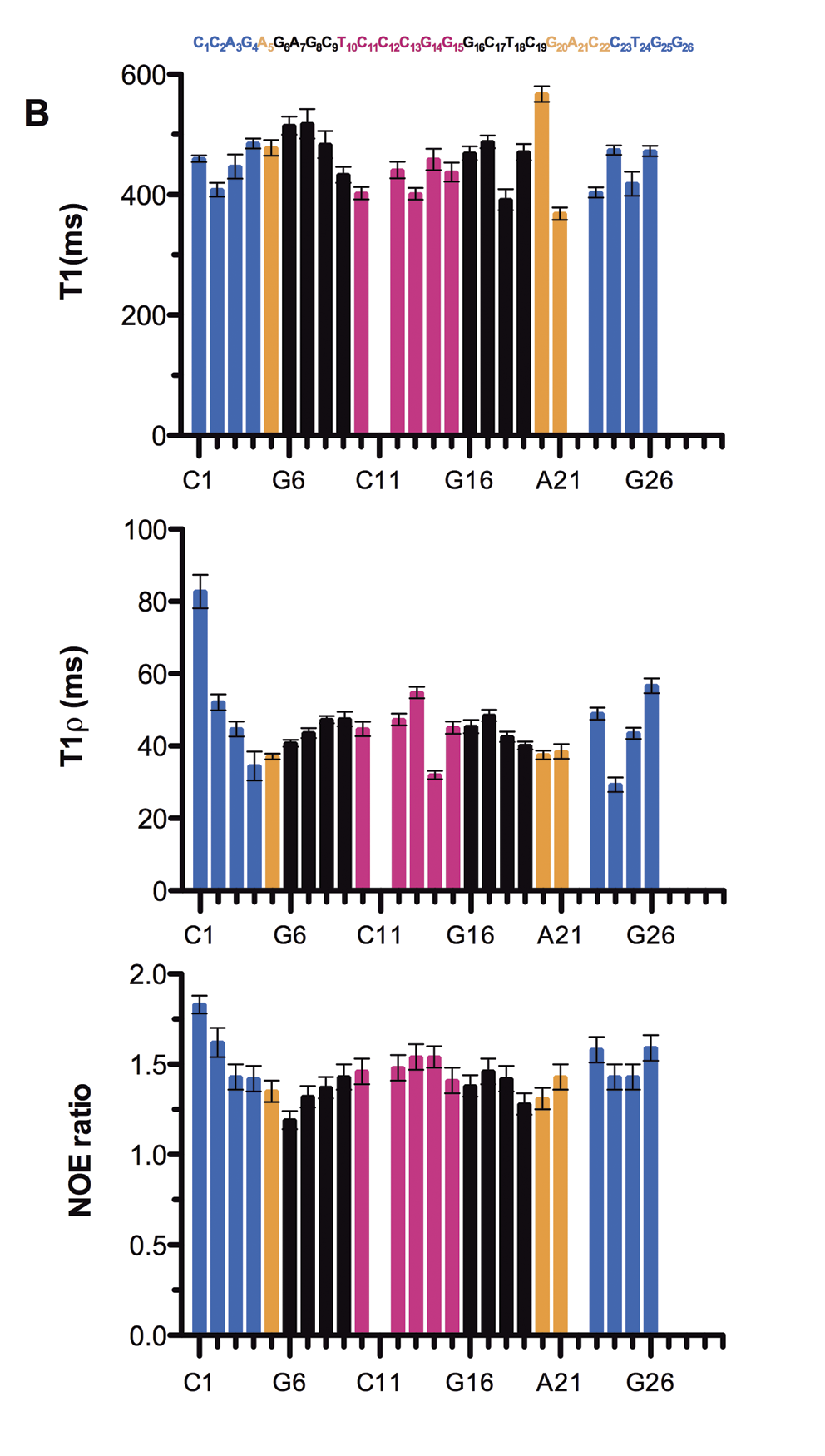

Supplement: Figure S2 — Relaxation times for anomeric C1′ carbons of mini-cTAR DNA at 500 MHz. spins. Top to bottom: 13C T1, 13C T1ρ and hetNOEs. Errors represent uncertainties in the fit of the primary relaxation data to mono-exponential decays. No data are associated to residues corresponding to a broad or overlapped cross peak. The color codes used for the residues are the following: blue (lower stem), orange (internal loop), black (upper stem) and magenta (apical loop). Comments of Figure 3 and Supplementary Figure S2: The profiles of T1, T1ρ and hetNOE values for both C1′ and C6/C8 carbons were found to be correlated with the sequence. The T1 profile for the successive parts of the molecule could be roughly described as: low (lower stem), high (upper stem), low (apical loop), high (upper stem), high (internal loop), low (lower stem). Note that for the T1ρ and hetNOE values, the profile is reversed. This profile is more apparent when only the central part (upper stem and apical loop) is considered. The data are therefore compatible with fast motions in the picosecond to nanosecond timescale for several residues of the apical loop. This kind of motion affects probably in an opposite way the T1 (decrease) and T1ρ/hetNOE (increase) values [44], [47]. Similar profiles for the various parameters of aromatic and C1′ carbons are indicative of coupled motions for base and sugar of the various residues of mini-cTAR [47]. (TIF) [file pone.0038905.s002.tif]

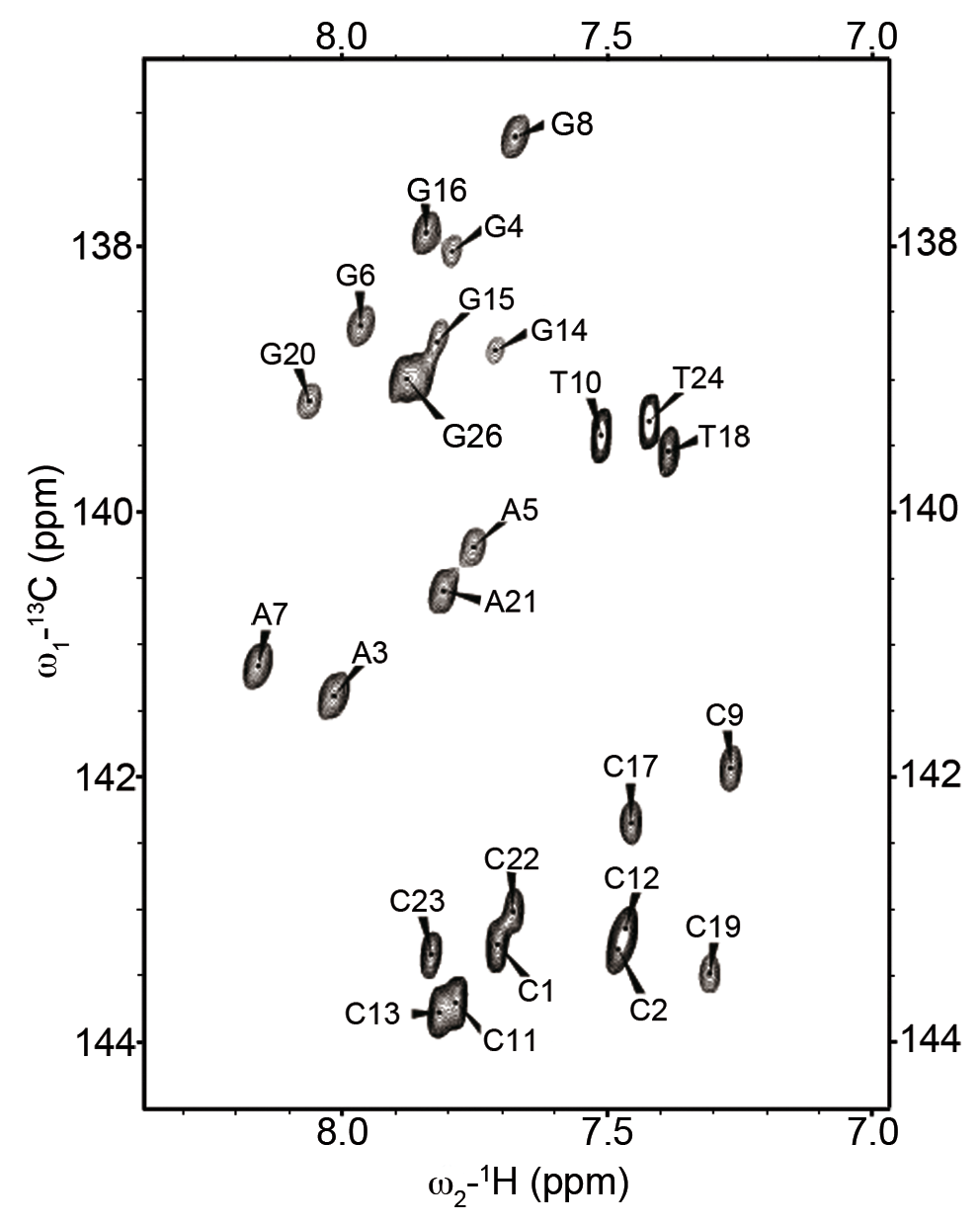

Supplement: Figure S3 — Region C8/C6–H8 of constant–time HSQC of 15N/13C labeled mini-cTAR DNA at 30°C. The cross peaks are indicated with the name of the corresponding residue. In this spectrum, G14 residue shows the broadest cross-peak among all residues of mini-cTAR (see text). (TIF) [file pone.0038905.s003.tif]
