# Supplementary material for: Intrinsic Nucleic Acid Dynamics Modulates HIV-1 Nucleocapsid Protein Binding to Its Targets
Source: PLoS One. 2012 Jun 20;7(6):e38905. doi: 10.1371/journal.pone.0038905 (PMC3380039; doi:10.1371/journal.pone.0038905)
Supplement: Materials S1 — Description of the procedure used in Quantitative Analysis of the 13C relaxation data. (DOC) [file pone.0038905.s004.doc]

***Materials S1***

***Description of the procedure used in Quantitative Analysis of the 13C relaxation data***

The estimation of the diffusion tensor parameters characterizing the global motion is a prerequisite to the quantitative analysis of internal motions. To determine these parameters, it is necessary to use the experimental *R_1_* and *R_2_* values of residues that are free of significant fast internal motions and slow conformational exchanges [1,2]. In consequence, fourteen *R_1_/R_2_* couples from aromatic and anomeric carbons of residues of the upper stem were retained while residues of the internal and apical loops as well as those of the lower stem were discarded. The global motion parameters were determined using a grid search by minimizing the difference between experimental and calculated values. A minimum is obtained with the following parameters for the diffusion tensor: (θ,φ) = (50°,140°), τ_c_=5.15 ns and *=*1.15. D*_II_* and are the parallel and perpendicular rotational diffusion constants of the axially symmetric diffusion tensor describing the overall motion and (θ,φ) are the polar angles that define the orientation of the unique axis (D*_II_* axis) of the diffusion tensor relative to the pdb frame of the molecular structure.

***References***

1. Tjandra N, Feller, S.E., Pastor, R.W., & Bax, A. (1995) Rotational diffusion anisotropy of human ubiquitin from 15N NMR relaxation. J Am Chem Soc 117: 12562-12566.

2. Oberstrass FC, Allain FH, Ravindranathan S (2008) Changes in dynamics of SRE-RNA on binding to the VTS1p-SAM domain studied by 13C NMR relaxation. J Am Chem Soc 130: 12007-12020.
